# Supplementary material for: Does digital inclusive finance promote industrial transformation? New evidence from 115 resource-based cities in China
Source: PLoS One. 2022 Aug 29;17(8):e0273680. doi: 10.1371/journal.pone.0273680 (PMC9423615; doi:10.1371/journal.pone.0273680)
Supplement: Attachment — Submitted filename: Plose one report.docx [file pone.0273680.s002.docx]

**Does digital inclusive finance promote industrial transformation? New evidence from 115 resource-based cities in China**

**Reviewer Comments:**

In the underlined research topic “Effect of COVID-induced Public Fear on European Stock Markets: A Proposal for an Algorithmic Trading System Based on Fear” is a novel part to the existing literature. The title of the article is remarkable; the presentation is good and the article may provide several scientific justifications.

- This material should be checked extensively to eliminate grammatical problems.
- I believe the author should provide more details on the study's limitations and future research.

Finally, it's a reputable effort; the theme is attractive and has several policy ramifications. Based on the explanation higher than, I recommend that the article be given "alteration" to construct it additional coherent earlier than it is "acceptance."
